# Commentary on: “The Presence of Active Brown Adipose Tissue Determines Cold-Induced Energy Expenditure and Oxylipin Profiles in Humans”

**DOI:** 10.1210/clinem/dgaa339

**Published:** 2020-06-02

**Authors:** Matthew D Lynes, Yu-Hua Tseng

**Affiliations:** 1 Section on Integrative Physiology and Metabolism, Joslin Diabetes Center, Harvard Medical School, Boston, Massachusetts; 2 Harvard Stem Cell Institute, Harvard University, Cambridge, Massachusetts

**Keywords:** human brown adipose tissue, thermogenesis, lipokines

Brown adipose tissue (BAT) is specialized for energy expenditure and consumes metabolic substrates in response to physiologic stimuli to increase body temperature. The most well understood stimulus for BAT activation is cold exposure, which increases sympathetic tone, resulting in both the activation of BAT and recruitment of new brown fat cells and the closely related beige adipocytes. Once activated, BAT takes up high levels of glucose and fatty acids to fuel a highly inefficient metabolic activity that wastes stored chemical energy as heat. In addition to dissipating energy, BAT also functions as a secretory organ, producing “BATokines” to affect metabolic functions in other tissues (1). Since the “rediscovery” of BAT in adult humans more than a decade ago, the potential to harness the high metabolic activity of this tissue as a means of increasing energy expenditure and combatting obesity has yet to be realized. Several factors have held back the translation of prospective therapeutics that could unleash the catabolic capacity of BAT.

The first limitation to leveraging BAT as a target for obesity therapeutics has been the lack of conclusive evidence that BAT in humans can play a significant role in energy balance. Although cold-activated BAT is recognized for its ability to act as a glucose sink, the impact of cold exposure on body weight has been nominal, partly due to the variable amounts of BAT present in human subjects. In a pioneering study, Yoneshiro et al first showed that cold can decrease adiposity only in subjects with detectable BAT as defined by [^18^F]-fluorodeoxyglucose ([^18^F]-FDG) uptake (2). Using a similar strategy to separate patients with and without BAT while controlling for body weight, Kulterer et al have effectively measured the effect of cold, which is known to specifically activate BAT thermogenesis, on whole-body energy expenditure in the presence or absence of BAT (3). Individuals with measurable BAT are able to increase energy expenditure in response to cold, while those lacking BAT cannot. These data provide direct evidence that cold-induced energy expenditure requires BAT. Interestingly, Kulterer et al have observed that subjects lacking BAT actually have elevated levels of norepinephrine in response to cold compared with individuals with BAT. These findings raise the possibility that people lacking BAT may have blunted signaling downstream of the sympathetic nervous system, and therefore, sympathomimetics may have limited effects in these individuals, precluding them from clinical studies that measure the effect of these drugs on BAT activity (4).

Although subjects with BAT have increased energy dissipation in response to cold simulation, there is no difference in energy expenditure between people with and without BAT at room temperature, which underscores the importance of activating BAT to maximize its effect on energy expenditure. Kulterer et al have calculated that, when fully activated, BAT-mediated energy expenditure is approximately 20 kCal over the 4 hours following a 90-minute cold exposure. Based on the same regimen of activation, 6 of the 90-minute cold exposures spaced out over the day could expend 120 kCal, which is consistent with other estimates of the capacity of this tissue (10-200 kcal/24 h) (5). The effect of different cold regimens on energy expenditure will need to be more thoroughly examined.

The second factor restraining the development of effective therapies that stimulate BAT to combat metabolic disease is the safe and efficient measurement of BAT volume and activity. As Kulterer et al rightly acknowledge, [^18^F]-FDG scanning is expensive and exposes subjects to radionuclides, making this approach difficult to apply to large groups of subjects or even perform repeatedly on the same individual. To address this limitation of human brown fat research, Kulterer et al have taken a biomarker approach to measure the concentrations of a panel of lipid molecules during cold exposure in both BAT-positive and BAT-negative individuals. These lipids are called lipokines, and they have been implicated in mediating organ crosstalk as well as cell signaling during thermogenic activation. In their study, Kulterer et al have identified a set of cytochrome P450 products, including 15d-PGJ2, 315_9.55 and 313_12.63, whose concentrations in circulation are all correlated with BAT [^18^F]-FDG uptake. Furthermore, they have validated the clinical observation that lipid diols, including 12,13-diHOME, 11,12-diHETre and diHOME_10.81 are all increased with cold, and interestingly observed a decrease in linoleic acid, which is the precursor for 12,13-diHOME (6). In addition, Kulterer et al have also found increased circulating levels of eicosapentaenoic acid and the 12-lipoxygense product 12-HEPE in subjects exposed to cold. Importantly, the changes of these cold-induced lipid mediators occur only in BAT-positive subjects, which is consistent with the notion that they are known as BATokines. Given the previously reported negative association of circulating levels of 12,13-diHOME and 12-HEPE with body weight and insulin resistance in humans, which Kulterer et al acknowledged, it would be interesting to study the effect of cold exposure on these clinical variates, especially in individuals with detectable BAT (6, 7).

The identification of these lipid biomarkers should facilitate the development of clinical studies to test different stimuli on BAT development and activity. In addition to their roles as biomarkers, the lipokines that were identified by Kulterer et al also have potential roles as effector molecules that could mediate the physiologic response to cold. This is clearly the case for 12,13-diHOME, which activates cellular fatty acid uptake, and also for 12-HEPE, which promotes glucose utilization and improves insulin sensitivity (6, 7). Further studies will be required to understand the effects of other lipokines identified in their screen.

While Kulterer et al are able to both provide convincing evidence that human BAT is able to respond to physiologic stimuli to increase energy expenditure, as well as identify a panel of biomarkers for safe and efficient measurement of BAT activity, challenges remain in the development of therapies that target this tissue. One interesting observation that Kulterer et al made was that the general variance in circulating lipid levels between individuals was larger than the variance between treatment groups. While they were still able to identify robust changes in certain lipid species in response to cold induced BAT activation, this suggests that lipidomic profiles are highly personal, even in a well-controlled clinical study with all study subjects fasting at least 10 hours overnight. Further research will be warranted to determine the effects of other biological variables, such as genetics and gender, on lipokine levels and to define baseline conditions that minimize interindividual variations, especially for lipokines used as clinically relevant biomarkers. Additionally, emerging research has begun to integrate genetic analysis with lipidomics based approaches to profile systemic metabolism in order to identify genetic targets that could be used to manipulate lipid levels (8).

We are excited that the study by Kulterer et al provides a premise to facilitate large clinical studies that target brown fat–mediated energy expenditure to treat metabolic disease. Lipokines identified by Kulterer et al in human subjects will require validation in larger cohorts, but they may also serve as interesting targets for preclinical development of novel treatments for obesity and the metabolic syndrome.

## Abbreviations

[^18^F]-FDG: [^18^F]-fluorodeoxyglucose
BAT: brown adipose tissue

## Additional Information

***Disclosure Summary:*** M.D.L. and Y.-H.T. are inventors on an US patent application related to 12,13-diHOME.

***Data Availability:*** Data sharing is not applicable to this article as no datasets were generated or analyzed during the current study.

## References

[1] Villarroya J, Cereijo R, Gavaldà-Navarro A, Peyrou M, Giralt M, Villarroya F New insights into the secretory functions of brown adipose tissue. J Endocrinol. 2019;243(2):R19-R27. 31419785 10.1530/JOE-19-0295

[2] Yoneshiro T, Aita S, Matsushita M, et al. Recruited brown adipose tissue as an antiobesity agent in humans. J Clin Invest. 2013;123(8):3404-3408. 23867622 10.1172/JCI67803 PMC3726164

[3] Kulterer OC, Niederstaetter L, Herz CT, et al. The presence of active brown adipose tissue determines cold-induced energy expenditure and oxylipin profiles in humans. J Clin Endocinol Metab. 2020;105(7):dgaa183. 10.1210/clinem/dgaa183 32343312

[4] O’Mara AE, Johnson JW, Linderman JD, et al. Chronic mirabegron treatment increases human brown fat, HDL cholesterol, and insulin sensitivity. J Clin Invest. 2020;130(5): 2209-2219. 31961826 10.1172/JCI131126 PMC7190915

[5] Carpentier AC, Blondin DP, Virtanen KA, Richard D, Haman F, Turcotte ÉE Brown adipose tissue energy metabolism in humans. Front Endocrinol (Lausanne). 2018;9:447. 30131768 10.3389/fendo.2018.00447 PMC6090055

[6] Lynes MD, Leiria LO, Lundh M, et al. The cold-induced lipokine 12,13-diHOME promotes fatty acid transport into brown adipose tissue. Nat Med. 2017;23(5):631-637. 28346411 10.1038/nm.4297 PMC5699924

[7] Leiria LO, Wang CH, Lynes MD, et al. 12-Lipoxygenase regulates cold adaptation and glucose metabolism by producing the Omega-3 Lipid 12-HEPE from brown fat. Cell Metab. 2019;30(4):768-783.e7. 31353262 10.1016/j.cmet.2019.07.001 PMC6774888

[8] Parker BL, Calkin AC, Seldin MM, et al. An integrative systems genetic analysis of mammalian lipid metabolism. Nature. 2019;567(7747):187-193. 30814737 10.1038/s41586-019-0984-y PMC6656374

